# Administration of Delphinidin to Improve Survival and Neurological Outcome in Mice After Cardiac Arrest and Resuscitation

**DOI:** 10.3390/antiox13121469

**Published:** 2024-11-29

**Authors:** Rika Bajorat, Stella Line Grest, Stefan Bergt, Felix Klawitter, Brigitte Vollmar, Daniel A. Reuter, Jörn Bajorat

**Affiliations:** 1 Department of Anesthesiology, Intensive Care Medicine and Pain Therapy, Rostock University Medical Center, Schillingallee 35, 18057 Rostock, Germany; 2 Department of Psychosomatic Medicine and Psychotherapy, Rostock University Medical Center, Goethestraße 18, 18055 Rostock, Germany; 3 Department of Anesthesiology and Intensive Care Medicine, Mediclin, 17192 Waren, Germany; 4 Institute of Experimental Surgery, Rostock University Medical Center, Schillingallee 69a, 18057 Rostock, Germany

**Keywords:** cardiac arrest, resuscitation, ischemia, reperfusion, oxidative stress, antioxidants, cerebral injury, delphinidin, anthocyanin

## Abstract

Reactive oxygen species (ROS) play an important role in ischemia–reperfusion (I/R) after cardiac arrest and cardiopulmonary resuscitation (CA-CPR). Early administration of vitamin C at a high dose in experimental models resulted in less myocardial damage and had a positive effect on survival after resuscitation. Here, we postulated that the ROS scavenging activity of an anthocyanin (i.e., delphinidin) would positively influence resuscitation outcomes. We hypothesized that administration of delphinidin immediately after CA-CPR could attenuate systemic inflammation in a standardized mouse model and thereby improve survival and long-term outcomes. Outcomes up to 28 days were evaluated in a control group (saline-treated) and a delphinidin-treated cohort. Survival, neurological and cognitive parameters were assessed. Post-CPR infusion of delphinidin deteriorated survival time after a 10 min CA. Survivors amongst the controls showed significantly more anxious behavior than in the pre-CPR phases. This tendency was also observed in the animals treated with delphinidin. In our study, we did not find an improvement in survival with delphinidin after CA-CPR and observed no effect on learning behavior. Our long-term behavioral tests clearly show that CA-CPR is associated with the development of post-interventional anxiety-like symptoms. Our findings open up scopes to investigate the intrinsic factors (e.g., oxidative stress, inflammatory and systemic-microbial response, etc.) influencing the therapeutic efficacy of anthocyanins in vivo.

## 1. Introduction

After successful cardiopulmonary resuscitation (CPR) following cardiac arrest (CA), patients often suffer from brain damage, myocardial dysfunction, aggravated by systemic ischemia–reperfusion injury, collectively known as post-cardiac arrest syndrome (PCAS) [1,2]. These primarily manifest with generalized activation of inflammatory reactions, which means that the clinical presentation is similar in many respects to that of sepsis or systemic inflammatory response syndrome (SIRS) [1,3]. The severity of ischemic injury sustained during cardiac arrest is directly proportional to the duration of the no-flow/low-flow state, whereas reperfusion injury is triggered by the rapid generation of reactive oxygen species (ROS) and recruitment of pro-inflammatory immune cells following restoration of circulation [4]. Increased ROS formation and systemic inflammation exacerbate organ damage due to disturbances in macro- and microcirculation (thrombogenesis in the small vessels), metabolic imbalances and direct leukocyte-mediated tissue destruction [1,5,6]. Despite reperfusion, the effects of precedent ischemia persist and continue to damage the tissue [6]. The pathophysiology of PCAS leads to further impairment of cerebral reperfusion and death of damaged neurons [7]. This primarily involves biochemical processes that can lead to neuronal death via the production of ROS and an increased release of excitatory neurotransmitters, i.e., glutamate [8]. Serum concentrations of various cytokines, soluble adhesion molecules and endotoxins are massively increased already three hours after the return of spontaneous circulation (ROSC). The severity of these changes correlates with the outcome [9]. Every year, around 95.9/100,000 adults worldwide suffer from CA outside of the hospital [10]. The prognosis is still very poor and the European population data show an in-hospital mortality of almost 90% of patients [11,12,13]. Among survivors, the neurological outcome in particular is largely limited. As a result, only less than 10% of patients return to a self-sufficient life [14,15,16].

ROS play an important role in ischemia–reperfusion (I/R) reactions after CA-CPR and also in other cardiovascular diseases [17]. ROS have a cytotoxic and pro-inflammatory effect on both, the endothelial cells and the neuronal cells [18]. Further, they directly activate the main transcription factors for the induction of pro-inflammatory cytokine expression NF-κB [19]. Superoxide (˙O2−) reacts easily with the cardio-protective agent nitric oxide (NO) and produces the highly cytotoxic peroxynitrite (ONOO−) [20], a cell-penetrating and powerful biological oxidant that reacts with DNA, membrane phospholipids, sulfhydryl groups and tyrosine. Furthermore, the ROS-mediated oxidation of critical lipids and low-density lipoprotein (LDL) in particular, is a key event in the pathogenesis of atherosclerosis [21]. ROS are also prothrombotic and promote platelet activation [22]. Hence, the elimination of ROS by antioxidants appears to be an attractive therapeutic option.

Early administration of high-dose vitamin C as an antioxidant resulted in less myocardial damage and showed positive effects on survival after resuscitation in experimental models of ventricular fibrillation cardiac arrest [23,24,25]. Experiments suggest that the underlying mechanisms are the inhibition of oxidative stress and the suppression of the transcription of inflammatory cytokines [25]. Treatment with high-dose vitamin C [200 mg/kg] 5 min after ROSC improved the survival rate and the neurological deficit score 72 h after restoration of spontaneous circulation compared to the animals in the control group [25]. Vitamin C administration therefore probably protects the integrity of the vascular endothelium. Vitamin C supplementation in patients with septic shock who have been resuscitated improves peripheral tissue perfusion and microvascular reactivity [26]. Idebenone, a free radical scavenger that can penetrate the blood–brain barrier, reduced oxidative brain stress after 7.5 min of cardiovascular arrest in rats [27]. Anthocyanins are also considered powerful antioxidants due to their poly-phenolic structure and are found in high amounts in a variety of berries, such as blackberries, blackcurrants, raspberries, red grapes, cranberries and elderberries [28]. Therefore, the use of a potent antioxidant in this group—such as delphinidin—might be beneficial. There is evidence that delphinidin has antioxidant properties and anti-inflammatory effects. In vitro, anthocyanins—including delphinidin—have consistently been shown to reduce ROS levels [29] along with diminished concentrations of pro-inflammatory cytokines (TNF-α, IL-6, IL-8, IL-1β) as well as with the expression of adhesion molecules (VCAM-1, ICAM-1, E-selectin, MCP-1) on endothelial cells [30]. Such anti-inflammatory effects of anthocyanins were also shown in vivo [30]. In addition, the inhibition of NF-κB and MAPK signaling pathways was demonstrated [30]. A potent suppression of COX-2 protein expression (central mediator of the inflammatory response) by delphinidin was demonstrated both in vivo and in vitro [31,32]. A delphinidin stabilized by sulfobutylether-β-cyclodextrin (CD) reduced inflammatory pain through both anti-oxidative and anti-inflammatory pathways in a model of hyperalgesia in rats [33]. Based on these findings, we hypothesized that the direct, systemic application of this stabilized delphinidin formulation could mitigate the development of a fulminant global inflammatory response in the early phase after resuscitation and therefore might inhibit PCAS. The primary focus of our investigation was to assess survival and neurological outcome after 10 min of cardiac arrest and subsequent cardiopulmonary resuscitation in a standardized mouse model [34,35,36,37,38]. For this purpose, we applied dedicated neurological, learning and behavioral tests to assess the long-term outcome [34,35,36,37].

## 2. Materials and Methods

### 2.1. Animals

Female wild-type mice (WT, *C57BL/6J*, n = 77) with a body weight of about 20 g and an age of about 4–5 months were used. The mice were held in a standardized environment (temperature-controlled environment of 22 °C, 12:12 h rhythm, free access to water and food). All procedures were conducted in accordance with national and international guidelines on the ethical use of animals (Council Directive 86/609/EEC of the European Communities). The experimental protocol was authorized by the Ethics Committee for the Care and Use of Laboratory Animals (local authority: State Office for Agriculture, Food Safety and Fisheries (LALLF) Mecklenburg-Vorpommern, approval number: LALLF M-V/TDS/7221.3-1-068/15). Every effort has been made to minimize animal suffering and to reduce the number of animals used. Since the study was designed as a pilot study, we assumed, based on the experience with this animal model and the survival rates from various previous studies [34,35,36,37], that a group size of n = 50 would result in at least 20 long-term survivors in each group.

### 2.2. Study Groups and Experimental Protocol

**Figure 1:**
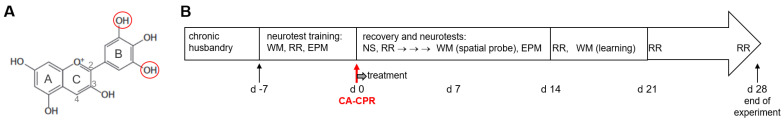
(**A**) The basic structure of delphinidin consists of a pyran ring (C) with a positively charged oxygen atom and a fused-on benzene ring (A), which together are also called benzopyran. A phenyl residue (B) is attached to position 2 of the pyran ring, whose substituents are specifically hydroxyl groups for delphinidin (labeled in red; adopted from Kern et al., 2007 [39]). In the glycosidic forms of anthocyanins, a sugar residue is usually bound to the hydroxyl group at position C-3 of the pyran ring via an O-glycosidic bond. (**B**) Experimental timeline. WM—water maze, RR—Rota Rod, EPM—elevated plus maze, NS—Neuroscore. Cardiac arrest was induced on day 0 and cardiopulmonary resuscitation was performed. Treatment with delphinidin was administered immediately after ROSC.

In the experiments, mice were randomly divided into two groups. All animals (study group and controls) received 0.2 mL saline solution as an intravenous infusion over one hour after ROSC. In the study group, delphinidin, freshly dissolved in saline, was added to that infusion. The anthocyanin delphinidin (Figure 1A) was a sulfobutylether-β-cyclodextrin (SEB-β-CD) stabilized water-soluble powder (Sapiotec GmbH, Würzburg, Germany; used in vivo (i.pl. or i.p. 2.5 mg/kgBW) and in vitro (10 µM) by Sauer et al. 2021 [33]). As a 2% solution, it contains 0.100 mg/mL of pure delphinidin. By this one-hour infusion, the animals of the study group received a total of delphinidin of 2.6 mg/kg. The experimental protocol is outlined in detail in Figure 1B. During the whole experimental time, the animals had free access to water and food, and no fasting protocol was used.

### 2.3. Anesthesia

All procedures except the behavior and learning tests were performed under general anesthesia. The mice were anesthetized by intraperitoneal injection of 12 µg/g ketamine /8 µg/g xylazine (10%; bela-pharm, Vechta, Germany/Rompun^®^ 2%; Bayer, Leverkusen, Germany). After immediate intubation using a 22-gauge cannula, the mice were mechanically ventilated with a FiO_2_ (fraction of inspired oxygen) of 0.2, a tidal volume of 10 µL/g and a respiratory rate of 120 breaths per minute (MiniVent, Hugo Sachs, March, Germany).

### 2.4. Cardiac Arrest and Resuscitation

The mouse CA-CPR model was performed as previously described [34,37]. Briefly, the anesthetized mice were placed on a self-regulating heating plate with continuous monitoring of body temperature using a rectal thermocouple probe (Effenberger, Pfaffingen, Germany) to prevent cooling. Monitoring and data acquisition of electrocardiography (needle probe; ECG; Animal bio Amp, ADInstruments, Bella Vista, New South Wales, Australia) and non-invasive blood pressure recording (NIBP controller, ADInstruments, NSW, Australia) was performed digitally (LabChart 5 pro, ADInstruments, NSW, Australia). CA was induced via a central venous catheter in the right jugular vein (PE50, ID 0.28 mm; Portex, Hythe, UK) by injection of 80 µg/g potassium chloride (KCl 7.45%; B. Braun Melsungen AG, Melsungen, Germany). After electrocardiographic verification of CA, mechanical ventilation was discontinued. After 10 min of CA, resuscitation was initiated. Precordial chest compression was started at a rate of 450/min using a modified sewing machine [40], 0.4 µg/g epinephrine (adrenaline 1:1000, InfectoPharm GmbH & Co. KG, Heppenheim, Germany) was injected and ventilation was resumed (220/min; FiO_2_ 1.0). After 2 min of CPR, the respiratory rate was reduced to 120/min and the FiO_2_ to 0.6. Twenty minutes after ROSC, the FiO_2_ was changed to 0.4 until extubation. After ROSC, all animals were reinfused by intravenous administration of 0.2 mL saline only (B. Braun Melsungen AG, Melsungen, Germany) or delphinidin solution for one hour.

### 2.5. Recovery and Well-Being Parameters of the Animals

Body weight was determined daily until 14 days after CA-CPR and then on days 21 and 28. In accordance with the guidelines for animal experiments, a loss of body weight greater than 30% from baseline was the criterion for withdrawing the animal from the experiment by administration of an overdose of i.p. injected ketamine/xylazine.

### 2.6. Neurological Assessment

All neurological and behavioral tests were assessed by a trained laboratory member who was blinded to the randomization of the animal groups.

*NeuroScore* is a modified grading score used for the standardized neurological assessment of mice, which includes the following issues: level of consciousness, corneal reflex, respiration, righting reflex, coordination and movement activity [34,37]. Animals that had not reached the maximum score of 12 points at the beginning of the study would have been excluded from the experiments. Immediately before CA-CPR, the NeuroScore was determined again for each animal. Within the first 24 h after the CA-CPR, the assessment was performed eight times, followed by a daily scoring for about 10 days and finally repeated on days 14, 21 and 28 after the CA-CPR (Figure 1B).

*Rota Rod*: All mice were trained to walk three repetitions on the Rota Rod for three consecutive days four days prior to CA-CPR to assess motor function, balance and coordination [34,35]. For one run, the maximum time on the rotating cylinder (12.5 rpm) was 300 s and the time from the start on the rod to the drop was measured. Rota Rod tests were carried out daily for ten days starting 24 h after CA-CPR and were repeated on days 14, 21 and 28 (Figure 1B).

*Water Maze:* The animals’ spatial learning and memory behavior were tested using the water maze with a hidden platform, as already described in detail [31]. In brief, a round basin (diameter of 90 cm), divided into four quadrants, was filled with room temperature water (approx. 23 °C) and a 5 × 5 cm platform was positioned invisibly just below the water surface in one of the four quadrants. Both the recording of the animals’ movements in the water maze and the subsequent analysis of the latency times until they reached the platform and the swimming distances were carried out using tracking software (EthoVision XT 11.5, Noldus, Wageningen, The Netherlands). All animals were allowed to habituate to the water maze without a platform the day before the learning task began (seven days before CA-CPR). For 5 consecutive days, all animals were trained to find the hidden platform, which was 0.5 cm below the water’s surface, so that the platform was invisible, and milk was added to the water. The animals were randomly placed at one of the 5 starting positions in the maze and had to find the hidden platform within 120 s. They completed 5 trials/day. If the platform was not reached within 120 s, the animal was manually placed on the platform and was allowed to rest and orient itself for 20 s. This was followed by another rest period of 20 s in a cage before the next trial began. After finished swimming, the animals were dried with a towel and warmed under a heat lamp. Spatial probe swimming for the memory test was performed twice with different starting positions (SW, SE) for 120 s without a platform after surviving resuscitation. To avoid the loss of animals by drowning due to general weakness in a catabolic state, all animals had to fulfill physical requirements such as a NeuroScore of 12, weight gain and 3 × 300 s on the Rota Rod [34,37]. Depending on individual recovery status, the spatial probe test was performed between days 7 and 14 (minimum-maximum) after CA-CPR (see Figure 1, light grey crosses). For the analysis of the spatial probe, the values of the two starts in the pool (SW, SE) were calculated separately and combined. The training then began again with the same protocol but with a different position (W) of the hidden platform in the maze.

*Elevated Plus Maze:* The elevated plus maze test (EPM) is an established method for measuring the fearfulness of rodents [41,42,43]. The test setup consisted of a cross at a height of 62 cm. Two opposite arms were enclosed by a 15 cm high wall (closed arms), the other two form-free bars in the room (open arms). The arms were 37 cm long and 6 cm wide. The 6 cm x 6 cm center area was located in the middle of the cross. The mice were placed at the junction of the four arms of the maze, facing an open arm. The entries/durations in each arm were recorded with a video tracking system (the same as used in the water maze: EthoVision) and simultaneously observed for 5 min by the experimenter. They underwent this test once before and once after the CA-CPR. Conventional parameters (total distance moved, time open arm, time closed arm, percent open arm time) and risk assessment parameters (stretch-attend posture, head dips, rears, grooming) were evaluated [43].

### 2.7. Statistics

Results are shown as mean ± standard deviation (SD) or mean ± standard error of the mean (SEM). All statistical analyses were calculated with SPSS version 27. At the end of the experiments, the number of surviving animals after CA-CPR was clearly below 20, so a non-normal distribution of the samples was assumed due to the small group sizes. Significance levels of the two groups, controls vs. delphinidin-treated, were calculated using the Mann–Whitney U test for independent samples and the Wilcoxon signed rank test for related samples, like comparisons within a group before and after CA-CPR. The survival of the mice was plotted using the Kaplan–Meier diagram and ranked according to the log-rank method (Mantel–Cox). The value of *p* ≤ 0.05 was defined as statistically significant. * is defined as ≤0.05 and *** <0.001.

## 3. Results

In total, 77 animals started the protocol. However, 12 animals did not complete the entire protocol because of different reasons (bleeding (n = 4), thromboembolism (n = 2), ventilation failure (n = 2), ROSC time greater than 120 s (n = 1), abandonment criteria according to the animal welfare (n = 3)). Finally, 65 animals were included in the analyses of the long-term observations. In total, 35 animals belonged to the control group and 30 animals to the study group.

### 3.1. Parameters of CA-CPR and Weaning

Values of heart rate, epinephrine dose and time to ROSC during CA-CPR are shown in Table 1. There were no differences in the baseline values before, during and immediately after resuscitation that can be controlled. We monitored these values between the control group and the intervention group so that these parameters could be excluded as the reason for possible differences. The heart rate in the group of mice that received delphinidin tended to be slightly higher than that of the control group and reached significance at 2 h after resuscitation (Table 1).

### 3.2. Recovery and Long-Term Survival After CA-CPR

**Figure 2:**
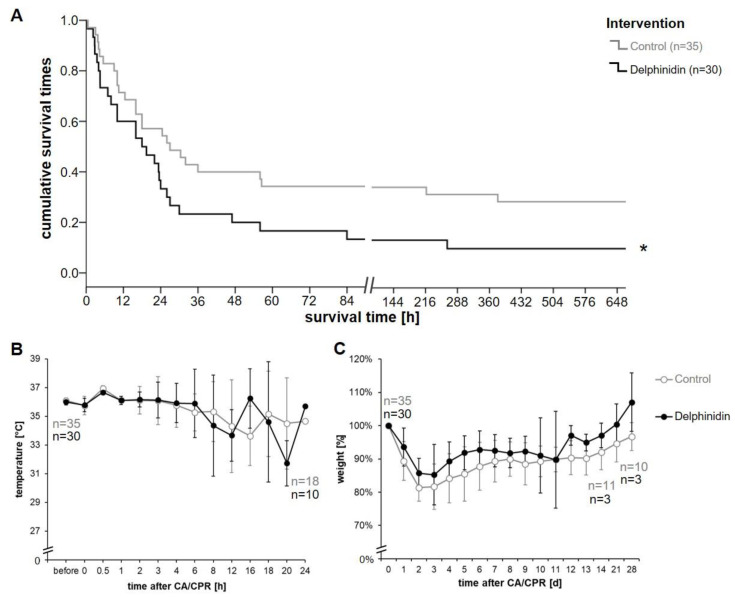
Survival, body temperature and development of body weight as recovery parameter after CA-CPR. (**A**) Kaplan–Meier plot for survival of mice after resuscitation following 10 min of CA within an observation period of 28 days. The mice that received delphinidin via the CVC immediately after ROSC had poorer survival than the control group (* *p* = 0.044 log-rank (Mantel–Cox)). (**B**) Monitoring of body temperature in °C within 24 h after CA (mean ± SD). (**C**) as a percentage of initial body weight on the day of cardiac arrest and resuscitation (mean ± SD).

In our long-term observational study, 28.6% (10/35) of the mice in the control group survived an induced a 10 min cardiac arrest and cardiopulmonary resuscitation with chest compressions, epinephrine and oxygen ventilation for 28 days. While most animals struggled to recover in the first 3 days, animals also died on days 9 and 16 after resuscitation, showing that our model at least partially mimics real-life situations. In the control group, 35% of the mice were still alive 72 h after resuscitation, but only around 18% in the delphinidin group. The survival curve of the animals that received delphinidin was well below that of the control animals (Figure 2A). Just under 10% of the delphinidin mice (3/30) survived the observation period of 28 days, which is significantly less than the survival rate (10/35) in the control group. Experiments were discontinued when it became clear that delphinidin treatment led to over-mortality. As a result, the study was terminated in the interests of animal welfare and the 3-R principle (Replacement, Reduction, Refinement). However, this also led to the consequence that further planned short-term experiments to investigate the mechanisms of action of delphinidin were not conducted.

The body temperature of the mice was monitored for 24 h after resuscitation and attempts were made to keep it at a minimum of 36 °C by applying additional heat. We wanted to exclude the influence of hypothermia on survival after resuscitation. There were variations, but no significant differences in this aspect between the groups (Figure 2B). The body weight of the test animals was initially recorded daily after resuscitation, and then weekly after a 14-day observation period as a parameter for well-being. We recognized a slighter decrease in body weight in the delphinidin group after resuscitation (approx. 15% of weight loss vs. controls almost about 20%), and the animals also appeared to gain weight better (Figure 2C), but differences between groups did not reach significance.

### 3.3. Neurological Assessment After CA-CPR

**Figure 3:**
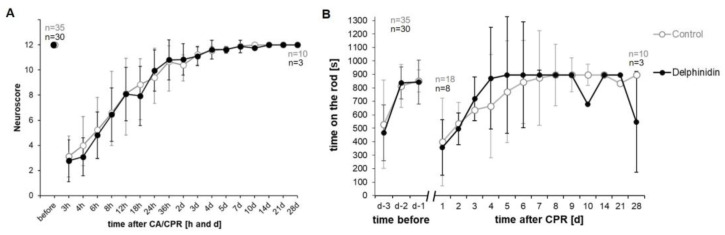
Neurological assessment over a 28-day observation period after CA-CPR. All graphs presented as mean ± SD. (**A**) The NeuroScore of the animals was almost identical between the intervention groups over the observation period. (**B**) The courses of RotaRod between the intervention groups according to CA-CPR did not differ over the observation period.

Although there were some remarkable deflections in the comparative curves of the RotaRod results on day 10 and day 28, all over the results of the neurological assessment by the Neuroscore and Rota Rod tests did not show any significant differences between both groups (Figure 3A,B).

### 3.4. Learning and Anxiety Behavior

**Figure 4:**
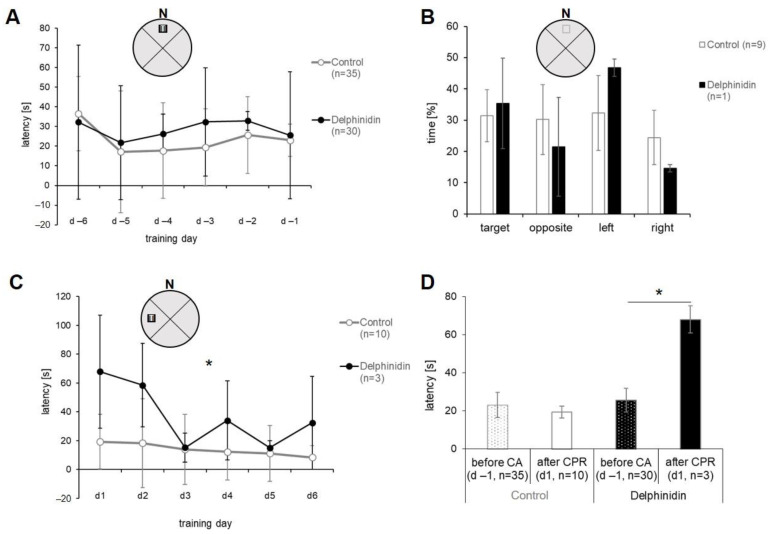
Water maze learning phase before CA (**A**) and spatial probe after surviving CA-CPR at the time of early recovery (**B**). The insets in (**A**–**C**) illustrate the water maze basin divided into four quadrants and the location of the invisible platform (T-Target). The course of the second learning phase with a changed location of the invisible platform (see inset) is shown in (**C**). It is tested whether the animals can retrain their spatial memory after surviving resuscitation. In (**D**), the latencies of the last day of the first learning phase (see in **A**) were compared with the latency of the first day of the second learning phase (see in **C**). All values were given in mean ± SD, except (**D**) in mean ± SEM. * marks statistical significance when *p* ≤ 0.05.

Latency in water maze refers to the time taken for a mouse to locate the escape platform. Figure 4A shows the first training and learning phase of all animals. Both groups displayed a learning effect from the first to the second day, which did not continue in the following days. The animals in the delphinidin group spent more time finding the underwater platform on training days 3 to 5 (Figure 4A: d-4, d-3 and d-2), but the difference was not significant at any time. The time level here on d-3 (32.3 ± 35.9 s) and d-2 (32.9 ± 39 s) was again at the starting level (d-6: 32.2 ± 24.5 s). The spatial examination (Figure 4B), which was performed after survival of resuscitation and initial recovery, showed no difference in the time spent in the target quadrant compared to the others. The result did not give any substantial information about the learning effect in the control group. In the delphinidin group, there were differences between the time in the target quadrant compared to the other three quadrants, but none were significant.

The relearning in the water maze using a different target quadrant with the invisible platform was started between the 7th and 14th day (see Figure 1B: experimental timeline), depending on the recovery status of the mouse, and lasted 6 days like the first learning cycle. Relearning curves after resuscitation were different in the individual groups. The delphinidin animals showed clear learning over the first 3 days (from latency 68 ± 39 s to 15 ± 10 s), which then stagnated but fluctuated until the end of the experiment. The control animals started with a latency of 19 ± 18 s and showed a continuously slightly flattening learning curve (Figure 4C). The control animals started with a time that could not become substantially faster. It seemed as if the mice remembered what to do. The results of relearning after surviving resuscitation tended to differ greatly between the groups, but only on day 4 did the difference reach significance (*p* = 0.036, Mann–Whitney U test; Figure 4C). When comparing the values from the end of the first learning training with the values from the beginning of the relearning training (Figure 4D), there was no difference in the controls, but there was a difference in the delphinidin-treated mice (*p* = 0.005, Wilcoxon sign-rank test).

**Figure 5:**
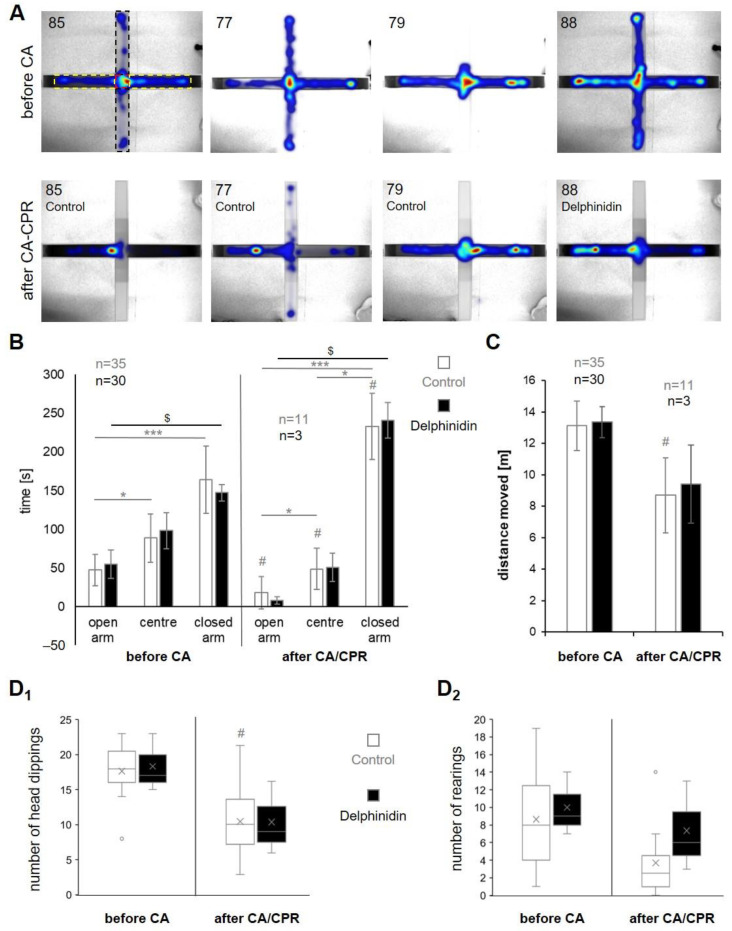
Elevated plus maze. All data except in C were given as mean ± SD. # Comparison within one group before and after CA-CPR using Wilcoxon Signed Rank test. Comparison within the * control group and the $ delphinidin group between different areas in the EPM arena (open arm, center, closed arm) carried out by two-factor analysis of variance for ranks according to Friedman for linked samples. (**A**) Examples of heatmaps before and after CA-CPR. Dashed lines mark the different areas of the arena: black—open arms, red—center, yellow—closed arms. (**B**) Time spent by the groups in the different areas of the EPM (open arms, center, closed arms). (**C**) The total distance moved by the mice in the EPM was presented as mean ± SEM. (**D**) Risk assessment. The counts how often the mice looked over the edge of the EPM (head dipping **D_1_**) and stretched up the walls of the closed arms, including standing on their hind legs (rearing **D_2_**). Head dippings were significant in the control group. *,#,$ marks statistical significance when *p* ≤ 0.05, *** *p* ≤ 0.001.

The exemplary heat maps of the EPM of four mice in Figure 5A showed that the mice in both groups behaved more “passive” or “anxious” after CA-CPR, but that from different starting levels and in different degrees. When comparing the control groups with the delphinidin-treated group, there were no differences in the behavior, meaning that the mice spent significantly more time in the closed arms after surviving resuscitation, regardless of the treatment (Figure 5B). After resuscitation, the time spent in the open arms and in the center of the EPM decreases and accordingly increases in the closed arms (# controls: open arms: *p* = 0.021, center: *p* = 0.016, closed arms: *p* = 0.008; delphinidin: before CA vs. after CA-CPR: *p* = 0.109 each region). Before the CA, the animals in both groups also spent longer times in the closed arms (* controls: open vs. center: *p* = 0.019, center vs. closed: *p* < 0.001, open vs. closed: *p* = 0.088; $ delphinidin: open vs. center: *p* = 0.683, center vs. closed: *p* = 0.102, open vs. closed: *p* = 0.041). This increased after resuscitation (* controls: open vs. center: *p* = 0.019, center vs. closed: *p* < 0.001, open vs. closed: *p* = 0.019; $ delphinidin: open vs. center: *p* = 0.221, center vs. closed: *p* = 0.221, open vs. closed: *p* = 0.014). The fact that all test animals generally moved less after surviving resuscitation is also shown in the total distance moved within the 5 min observation period, whereby the values were only significant in the control group (before vs. after CA-CPR #: *p* = 0.008; delphinidin: *p* = 0.109; Figure 5C). Presumably, the differences within the delphinidin group did not reach the significance level. The results of the frequency of head dipping confirmed this, especially for the control animals (before vs. after CA-CPR #: *p* = 0.019, Figure 5D_1_), whereas the delphinidin group tended to show the same but did not reach significance. There was no difference in the number of rearings between before and after CA-CPR (Figure 5D_2_), even if the control groups tended to look the opposite (*p* = 0.083).

## 4. Discussion

In the present study, the antioxidant delphinidin was tested in a mouse model of CA-CPR to attenuate the activation of the immune response by ROS reduction, with the focus of the investigations being on long-term survival and neurological outcome. However, the delphinidin-treated mice had no advantage in long-term survival after resuscitation, but rather the opposite. The temperature fluctuations seen in Figure 2B between 8 and 24 h after CA-CPR can be explained by two things: First, by trying to control the body temperature and prevent the surviving mice from cooling in no or low activity state in the first 24 h after resuscitation, they were observed in a terrarium with a temperature of 28–30 °C. This temperature could not be optimized for every individual animal. On the other hand, body temperature was measured non-invasively using a thermal imaging camera. This was carried out on a 1 cm^2^ piece of hairless skin on the ventral side, which became more difficult depending on the animals’ level of alertness. Two hours after resuscitation, the heart rate of the mice in the delphinidin study group was higher than that of the control group, which may already be an indication of a problem. The epinephrine dose during resuscitation was the same in the comparison groups, so a higher heart rate could not be attributed to this. The excess mortality depicted a detrimental effect of delphinidin, in contrast to our expectations. As the disbalance of pro- and anti-oxidants after CA in the plasma begins at an early phase after resuscitation [44], it seemed reasonable to administer the antioxidant as early as possible after the event. Anthocyanins from berries are usually present in the more stable glycosylated form, but after consumption, they undergo digestion processes that break down the sugar component [45]. As the mice were still under anesthesia after successful resuscitation and some were still unconscious after anesthesia, oral administration of delphinidin was not feasible. We used a stabilized formulation of delphinidin for systemic post-CPR application in our model. In our opinion, this pattern of administration is closest to a possible clinical application of antioxidative compounds. However, we did not analyze the plasma concentrations of delphinidin or its degradation products. Sauer et al. used exactly the same delphinidin in vivo as we did. They applied it intraplanar into a painfully inflamed hind paw in rats and also intravenously to compare the effect [33]. Both types of application showed a decrease in delphinidin in the plasma approximately by 2/3 and in the tissue almost completely after 60 min [33]. Under physiological conditions, sugar-free anthocyanins may further metabolize into smaller phenolic compounds, e.g., phenolic acids and aldehydes [39,46,47]. In other in vivo studies on rats and humans, orally administered pure delphinidin 3-O-β-rutinoside, delphinidin 3-O-β-glucoside and equivalent cyanidin-forms were detected in the intact form in plasma at different time points after consumption [48]. While the exact form in which these are absorbed and present in the bloodstream may differ, it is possible that the degradation products (simple phenolic acids and/or aldehydes) predominate over the actual compound [17,46]. This is a relevant detail, as phenolic compounds can paradoxically have pro-oxidant properties [49,50]. That could possibly explain the observed higher mortality in the delphinidin group. This raises the concern of how the uptake of the parent polyphenols can exert a direct antioxidant effect in vivo. There are studies that attribute the protective role of flavonoids and polyphenolic compounds in heart failure to their ability to scavenge oxygen free radicals, inhibit the inflammatory process and endothelial dysfunction, maintain NO levels, and inhibit mast cell secretion [51,52]. These compounds are highly effective in directly scavenging peroxyl radicals and in reducing oxidative stress in the heart, as evidenced by reduced malondialdehyde formation [51,52]. However, an in vitro model of I/R using cultured rat embryonic ventricular H9c2 cells compared a wide range of flavonoids including delphinidin for their protective effects against cardiac ischemia–reperfusion injury [53]. The concentrations were those that could be achieved in plasma with acute or long-term dietary supplementation (5–10 μM at high levels) [53]. A three-day (long-term) pre-treatment with different concentrations of flavonoids showed little to no protection, whereas a short-term treatment (one hour) with delphinidin showed a protection of about 10% in the cell culture [53]. At higher concentrations, delphinidin (≥50 µM) showed significant cytotoxicity, while lower concentrations did not [53,54]. Delphinidin (40 µM) can protect human chondrocytes in vitro from oxidative stress by regulating Nrf2 and NF-B and activating autophagy [54]. At higher concentrations, delphinidin affects the cellular protective mechanisms by inhibiting autophagy so that ROS can accumulate and ultimately cause apoptosis in human-derived osteosarcoma cells in vitro [55]. Cytotoxic effects could be an explanation for the significantly worse post-resuscitation survival of the mice treated with delphinidin in our study; however, we did not administer a comparable dosage. Metabolites may have formed from delphinidin after infusion, which could have aggravated the damage to the endothelial tissue through their pro-oxidative effect [49]. Another point is that the majority of the in vitro experiments were pretreated with delphinidin or other anthocyanins for up to 3 days before oxidative stress induction [53,55,56]. In order to mirror the clinical reality of sudden CA in our mouse model, treatment approaches were always initiated at the time of resuscitation and not earlier. An in vivo study on cardiac hypertrophy with intraperitoneal administration of delphinidin did not show such effects [57]. Mice were subjected to transverse aortic constriction (TAC) and, after three days of recovery, received peritoneal injections of delphinidin dissolved in DMSO at a concentration of 5 or 15 mg/kg/day for 8 weeks [57]. Eight weeks after TAC surgery, marked myocyte hypertrophy was observed, as evidenced by the increased cross-sectional area of myocytes compared to the sham control group, accompanied by increased ROS levels and NADPH oxidase activity. However, these changes were attenuated by delphinidin at the high dose (15 mg/kg/day), while no significant changes were observed under a low dose (5 mg/kg/day) [57]. In our study, 2.6 mg/kg delphinidin was infused intravenously over a period of one hour, which was still below the daily dose described by Chen and colleagues [54], which showed no effect. At a daily dose of 15 mg/kg over 8 weeks, delphinidin had almost no toxic effects or side effects on the heart, liver, lungs and kidneys [57]. Although the mode of conduct was different, we doubted that the observed excess mortality in our model could be caused by that. Since ROS trigger oxidative stress during inflammation, have chemotractive effects and can promote the migration of immune cells, an increase in vivo in 4-hydroxy-2-nonenal (4-HNE, ROS-marker), a secondary product of ROS from lipid peroxidation, VCAM-1 in endothelial cells and macrophage infiltration was observed [33]. Treatment with delphinidin lowered 4-HNE levels, reduced the number of infiltrating macrophages and downregulated the expression of VCAM-1. The authors consider a double effect to be plausible: delphinidin scavenges ROS and cyclodextrin captures 4-HNE [33]. However, these promising results could not be translated into an improved outcome in our resuscitation model, mimicking a real-life application.

In our study, we did not detect any influence on learning behavior in the water maze task. Our long-term behavioral tests clearly show that CA-CPR increases anxiety-like behavior. In the elevated plus maze, the untreated mice (controls) spent significantly more time in the closed arms and less time in the open areas, the number of head dippings decreased and they generally moved less. This can also be observed in the delphinidin-treated mice but did not reach significance due to the small number of survivors after CA-CPR. Neigh et al. found that the behavioral deficits were related to brain injury following untreated CPR, specifically due to damage at the CA1 region of the hippocampus [58]. Elevated anxiety levels may be responsible for the difficulties reported by the clinical population in concentrating, interacting with others and maintaining employment [59,60,61]. Almost a quarter of cardiac arrest survivors suffer from psychiatric comorbidities, where mood disorders (including depression) are the most frequent, followed by anxiety [61]. In addition, underlying anxiety may be responsible for depressive symptoms in CA-CPR survivors.

It is not certain whether the result would have been different with another delphinidin dosage or its mode of administration. It is noteworthy that natural compounds such as delphinidin often undergo complex processes in human physiology and their effects may depend on various factors. Over many years, the health benefits of anthocyanins have been widely demonstrated [28,57], but the mechanism of action and clinical benefits do not appear to be entirely clear. Nevertheless, there is a remarkable inter- and intra-variability in the bioavailability of anthocyanins due to several factors such as the food matrix or technological/processing conditions, enzymatic patterns, and microbiome composition [62]. In order to be able to use anthocyanins, e.g., delphinidin as medication, their metabolic mechanisms and effects need to be understood in explicit detail.

The strength of the present study is the use of the well-established resuscitation model, which was orientated to mimic a real-life clinical emergency and pathway of rehabilitation. The focus was on long-term survival and neurological outcomes verified by behavioral, learning and neurological tests, which are essential for CA-CPR survivors. However, some limitations have to be mentioned. Due to the higher mortality in the delphinidin group, it was decided not to continue the long-term experiments (28 days after CA-CPR) up to the originally planned group size in favor of animal welfare. Therefore, there are no short-term data available on blood and organ recovery in the first hours and up to 3 days after CA-CPR. Furthermore, as no molecular and protein analysis was performed in the present study, we can neither determine the level of inflammation or ROS production within the animals nor conclude about the exact actions of delphinidin at the cellular level. Based on the fact that there was no described toxic effect in the range of the concentration we used in our study, it was surprising to see the clinical outcome presented. Since the clinical interest focuses on improving the long-term neurological outcome after resuscitation, we believe that the study design to focus first on survival and outcome is appropriate. Previous studies using our resuscitation model have confirmed this approach [34,35,36,37].

*p*-value adjustment for multiple comparisons (e.g., a Bonferroni correction) was not performed as only three animals in the delphinidin group survived the 28-day observational period. Therefore, some comparisons in the behavioral and learning tests must be evaluated with statistical caution due to the small group size.

## 5. Conclusions

In contrast to the general expectation, delphinidin did not improve but decreased survival after CA-CPR in a well-established mouse model. Anxiety was increased in resuscitated animals, irrespective of the administration of delphinidin. A clinical use of delphinidin in this clinical context is not recommended. Further in-depth studies on metabolism, molecular pathways and biovariability seem to be essential before anthocyanins like delphinidin are used in complex clinical models in the future.

## Figures and Tables

**Table 1:** Basic parameters of CA-CPR and weaning. The significance levels were calculated using the Mann–Whitney U test for independent samples.

Experimental Groups	Control (Saline)	Delphinidin	
**Parameter**	Mean ± SD	Mean ± SD	***p*** **Value**
number:	(n = 35)	(n = 30)	
**Baseline before CA**			
Body weight [g]	20.09 ± 1.05	20.22 ± 1.16	0.906
heart rate [1/min]	213 ± 29	224 ± 57	0.727
MAP [mmHg]	78.16 ±12.62	78.93 ± 15.00	0.925
body temperature [°C]	36.09 ± 0.76	36.0 ± 0.3	0.878
**CA**			
dosage epinephrine [µg]	13.56 ± 4.28	12.42 ± 3.04	0.456
time to ROSC [sec]	66.26 ± 29.31	61.33 ± 21.49	0.746
Extubation [min]	175.76 ± 20.79	173.96 ± 25.68	0.595
**1 h after CA**			
	(n = 34)	(n = 29)	
heart rate [1/min]	352 ± 78	377 ± 93	0.258
MAP [mmHg]	61.00 ± 10.10	67.45 ± 12.34	0.076
body temperature [°C]	36.1 ± 0.2	36.1 ± 0.18	0.428
**2 h after CA**			
	(n = 34)	(n = 26)	
heart rate [1/min]	280 ± 93	339 ± 100	0.037 *
MAP [mmHg]	60.50 ± 8.64	66.0 ± 10.80	0.270
body temperature [°C]	36.1 ± 0.16	36.2 ± 0.28	0.961
**3 h after CA**			
	(n = 24)	(n = 18)	
heart rate [1/min]	354 ± 96	368 ± 111	0.542
MAP [mmHg]	63.33 ± 7.64	73.33 ± 10.41	0.400
body temperature [°C]	36.1 ± 0.96	36.1 ± 0.52	0.400

* marks statistical significance when *p* ≤ 0.05.

## Data Availability

The original research data will be shared on request.
